# An affordable and convenient diagnostic marker to identify male and female hop plants

**DOI:** 10.1093/g3journal/jkad216

**Published:** 2023-11-14

**Authors:** Shaun J Clare, Ryan M King, Anna L Tawril, Joshua S Havill, Gary J Muehlbauer, Sarah B Carey, Alex Harkess, Nahla Bassil, Kayla R Altendorf

**Affiliations:** National Clonal Germplasm Repository, USDA-ARS, 33447 Peoria Road, Corvallis, OR 97333, USA; National Clonal Germplasm Repository, USDA-ARS, 33447 Peoria Road, Corvallis, OR 97333, USA; Forage Seed and Cereal Research Unit, USDA-ARS, 24106 N Bunn Road, Prosser, WA 99350, USA; Department of Agronomy and Plant Genetics, University of Minnesota, 1991 Upper Buford Circle, St.Paul, MN 55108, USA; Department of Agronomy and Plant Genetics, University of Minnesota, 1991 Upper Buford Circle, St.Paul, MN 55108, USA; HudsonAlpha Institute for Biotechnology, 601 Genome Way Northwest, Huntsville, AL 35806, USA; HudsonAlpha Institute for Biotechnology, 601 Genome Way Northwest, Huntsville, AL 35806, USA; National Clonal Germplasm Repository, USDA-ARS, 33447 Peoria Road, Corvallis, OR 97333, USA; Forage Seed and Cereal Research Unit, USDA-ARS, 24106 N Bunn Road, Prosser, WA 99350, USA

**Keywords:** hops, *Humulus lupulus*, sex determination, diagnostic marker, association mapping, Plant Genetics and Genomics

## Abstract

Hop production utilizes exclusively female plants, whereas male plants only serve to generate novel variation within breeding programs through crossing. Currently, hop lacks a rapid and accurate diagnostic marker to determine whether plants are male or female. Without a diagnostic marker, breeding programs may take 1–2 years to determine the sex of new seedlings. Previous research on sex-linked markers was restricted to specific populations or breeding programs and therefore had limited transferability or suffered from low scalability. A large collection of 765 hop genotypes with known sex phenotypes, genotyping-by-sequencing, and genome-wide association mapping revealed a highly significant marker on the sex chromosome (LOD score = 208.7) that predicted sex within our population with 96.2% accuracy. In this study, we developed a PCR allele competitive extension (PACE) assay for the diagnostic SNP and tested three quick DNA extraction methodologies for rapid, high-throughput genotyping. Additionally, the marker was validated in a separate population of 94 individuals from 15 families from the USDA-ARS hop breeding program in Prosser, WA with 96% accuracy. This diagnostic marker is located in a gene predicted to encode the basic helix-loop-helix transcription factor protein, a family of proteins that have been previously implicated in male sterility in a variety of plant species, which may indicate a role in determining hop sex. The marker is diagnostic, accurate, affordable, and highly scalable and has the potential to improve efficiency in hop breeding.

## Introduction

Hop (*Humulus lupulus* L.) of the Cannabaceae family is a key ingredient in beer production, providing aroma, bitterness, and preservatives to the final beer product (Lafontaine *et al*. 2019). Currently, 5 subspecies or botanical varieties of hops are recognized, namely *lupulus*, *lupuloides*, *neomexicanus*, *pubescens*, and *cordifolius*, of which *lupuloides*, *neomexicanus*, and *pubescens* are native to North America (McCallum *et al*. 2019). Hops are worth an estimated $620 million in raw commodity value in the United States (USDA NASS 2022). The United States is the largest producer of hops globally, with 101 million lbs across ∼60,000 acres in 2022 in Washington (71% of production), Idaho (16%), and Oregon (13%) (USDA NASS 2022). Hop is a dioecious, perennial, wind-pollinated species with a diploid genome (2n = 18 + XX/XY) that is ∼3 Gb in size (Padgitt-Cobb *et al*. 2023). Cones from female plants are used in brewing and are grown commercially, whereas males are used almost exclusively in breeding (Paguet *et al*. 2022). Male hop plants can become established in commercial yards from shattered, open-pollinated seeds, but they are routinely culled to prevent seed production, which can be associated with a reduction in hop quality (Thomas and Neve 1976). Hop breeding is typically conducted using one or a combination of the following methods: mass selection, hybridization (pedigree based or open pollinated), and mutation or polyploid breeding (Haunold 1981). Breeding objectives include resistance to downy and powdery mildews, improvements in yield, unique aroma and brewing characteristics, and increases in alpha acid. The hop variety development timeline is long, ranging from 10 to 15 years (Haunold 1981), which makes it difficult for breeders to keep up to changing trends in brewing, as well as evolving disease pressures, and temperature and weather extremes (Paguet *et al*. 2022). While there has been considerable effort to identify molecular markers in hop in the hope of improving breeding efficiency for traits such as chemical characteristics (Koie *et al*. 2005; Čerenak *et al*. 2006, 2009; Patzak *et al*. 2012; McAdam *et al*. 2013), disease resistance or susceptibility (Seigner *et al*. 2009; Henning *et al*. 2011, 2015, Henning, Gent, *et al*. 2017; Padgitt-Cobb *et al*. 2019; Havill *et al*. 2023; Olatoye *et al*. 2023), and plant stature (Henning, Hill, *et al*. 2017), there has been limited routine application of marker-assisted selection (MAS) or genomic selection in published literature, and historically, varieties have been developed exclusively through phenotypic selection.

In some regions, hop plants do not become reproductively mature within the first year after transplanting. In regions where they do, their yield and quality may not be fully representative of established plantings, which mandates multiple years of evaluation (Donner *et al*. 2020). Thus, each time a hop is transplanted to a different evaluation stage of the breeding cycle (i.e. seedling to single hill to advanced, multi-hill plot), it can add another year to the development timeline. A knowledge of seedling sex prior to planting may allow the breeder to strategize, depending on the objective of the cross, by optimizing the number of individuals of each sex that get planted and evaluated and the location of planting, thus enabling variable selection intensity for traits earlier in the breeding cycle. In addition, breeders may choose to create a female-only seedling evaluation yard, which would exclude pollen and result in improved cone quality for phenotypic selection. In a traditional hop breeding program in the United States, the first year of the cycle is spent distinguishing between male and female plants. Depending on the program or goal, males may be discarded and females transferred to another field or expanded for further evaluation. Therefore, perhaps the utmost interest and progress seen in developing resources for MAS techniques in hop has been in the development of a marker to distinguish male and female hop at an earlier stage in the breeding process (Prentout *et al*. 2021).

Hop sex is determined by using an XY system, whereby accessions with XX are female and XY are males (Parker and Clark 1991; Shephard *et al*. 2000). However, monoecious accessions commonly occur, especially under stress conditions, with genetic mechanisms yet to be discovered. A collection of previously developed sex-linked markers has included random amplified polymorphic DNA (Polley *et al*. 1997; Buck *et al*. 2009), inter-simple sequence repeat (SSR) (Danilova and Karlov 2006), SSR (Jakse *et al*. 2008), and cytogenetic markers (Divashuk *et al*. 2011). However, these have primarily been conducted in local biparental populations, and the resulting marker has been located on the male chromosome Y. These markers have, therefore, been dominant presence/absence markers of limited value outside the biparental population. The SSR marker HLAGA7 has been shown to be completely linked with hop sex; however, this has been largely performed in biparental populations with Slovenian pedigreed hop plants (Jakse *et al*. 2008; McAdam *et al*. 2013), and therefore, not validated in more diverse germplasm. The HLAGA7 SSR was also included in a fingerprinting set developed for hop accessions (Driskill *et al*. 2022). The most recent advancement was the development of a multiplex set of 4 SSRs that show 100% accuracy in 97 cultivated hops and 2 populations with Slovenian heritage and 1 population of US origin (Čerenak *et al*. 2019). This 4-SSR multiplex was validated in 14 populations within the Slovenian breeding program and it therefore demonstrated wider transferability, but it has not been validated in a wider variety of paternal sources. In addition, the SSR multiplex is gel based, and the results require human interpretation, ultimately leading to a low-throughput and high labor–intensity assay when considering other molecular approaches. Fluorescent competitive allele-specific primer approaches are reported to be 45 times faster than gel-based systems (Rasheed *et al*. 2017) and can be automatically called at superior throughput. We, therefore, sought to develop a low-cost, high-throughput marker from a diverse set of germplasm that could be utilized to distinguish males and females in the early stages of hop breeding to increase efficiency. Our focus was not to explore the genetics of sex determination but to develop, validate, and employ a marker that is accurate, practical, and of low cost for identifying sex in large populations of hop seedlings.

## Materials and methods

### Plant materials

A diverse collection of male and female hop germplasm was assembled from the USDA-ARS National Clonal Germplasm Repository (NCGR) in Corvallis, OR, the USDA-ARS hop breeding program in Corvallis, OR, the Clean Plant Center Northwest in Prosser, WA, and the former Washington State University (WSU) breeding program in Prosser, WA. A total of 1,152 genotypes were grown in a greenhouse setting, and 50 mg of young leaf tissue was collected, flash-frozen at −80°C, lyophilized using a Freezone 4.5L −84°C Benchtop Freeze Dryer (Labconco, Kansas City, MO, USA) at −80°C and 0.2 mbar for 48 h, and sent to the Center for Qualitative Life Sciences (CQLS) at Oregon State University (OSU) for DNA extraction using the Omega Biotek kit M1130 (MagBind Plant DNA DS, Norcross, GA, USA). Data for plant sex in this collection were assembled by referencing germplasm releases, passport information from the National Plant Germplasm System, and breeding program records (see Supplementary File 1).

The validation population was composed of seedlings derived from 15 independent crosses made in 2021 at the USDA-ARS hop breeding program in Prosser, WA. Seeds were threshed from cones manually using sieves and a grout float and were separated from chaff using a South Dakota Seed Blower (Seedburo, Des Plaines, IL, USA). The seeds were then sterilized and stratified according to Haunold and Zimmermann (1974). Established seedlings in 5 cm pots were transplanted into a seedling yard (3 m row spacing, 2.4 m trellis height) at 0.45 m spacing at the WSU-Irrigated Agriculture Research and Extension Center (WSU-IAREC) in Prosser, WA, in early June 2022. Plants were irrigated and fertilized, and weeds were cultivated as needed. The plants were strung using a single strand of 24-ply cotton twine, and a single bine per plant was trained in mid-June 2022. Once plants reached 50% up the trellis, basal growth was trimmed using scissors to promote reproductive growth. Phenotypic data were collected for plant sex by evaluating flower morphology on mature plants. Ninety-four plants with known sex phenotypes were selected and propagated via rhizomes in the greenhouse, where young leaf tissue was collected, flash-frozen, and lyophilized as described (Supplementary File 2). An additional 299 hop accessions obtained from Havill *et al*. (2023) were used for further validation (Supplementary File 2).

### Sequencing and data curation

Genotyping-by-sequencing libraries were developed for 12 plates using the ApeKI enzyme (Elshire *et al.* 2011) at CQLS at OSU. Libraries were sequenced on an Illumina NextSeq 2000 with 96 samples per P2 cell with 100 bp single-end reads. Variant calling was performed using a custom pipeline (Supplementary File 3). In brief, fastq files were demultiplexed using *flexbar 3.5.0* (Roehr *et al*. 2017) into compressed sample fastq files. Reads were trimmed for adapters and quality-filtered using *fastp 0.22.0* (Chen *et al*. 2018) by providing the adapter sequence, removing the first and last 10 nucleotides, low-complexity regions, right cut sliding window for quality falling below 20, and a read length of at least 50 nucleotides. The number of first and last nucleotides to remove was determined using *fastqc 0.11.9* (Andrews 2010) and *multiqc 1.12* (Ewels *et al*. 2016). Sample reads were aligned to the indexed reference genome of “Cascade” (Padgitt-Cobb *et al*. 2023) from HopBase (Hill *et al*. 2017) using a custom piping loop with *bwa-mem2 2.2.1* (Vasimuddin *et al*. 2019) and *samtools* (Li *et al*. 2009) to generate sorted bam and index files. Variant calling was performed on individual samples using *HaplotypeCaller* within *GATK 4.2.1.6* (Poplin *et al*. 2018) for gvcf files. The gvcf files were reblocked with *ReblockGVCF* before being imported into a *GenomicsDB* database using *GenomicsDBImport* for each individual chromosome using a custom bash script on the OSU cluster. Joint variant calling was performed on each chromosome using *GenotypeGVCF* and a custom bash script on the OSU cluster. Each chromosome joint calling vcf file was merged into a single genotyping file using *MergeVcfs*. The vcf file was filtered using *vcftools 0.1.16* (Danecek *et al*. 2011) for biallelic SNPs, minimum quality scores of 30, and minimum depth of 3 before iteratively filtering down to missing data of 30% for both variants and samples to maximize data retention. Finally, variants were filtered for a minor allele frequency of 0.05 and thinned to 100 bp. The remaining missing data were imputed using *Beagle 5.2* (Pook *et al*. 2020) using a burn-in of 10 and 50 iterations (Supplementary File 4).

### Association mapping, haplotyping, and candidate gene assessment

Samples with inadequate amounts of sequencing data, unconfirmed phenotypes, or replicated samples were removed from the analysis. A total of 765 individuals (Supplementary File 1) and 20,861 biallelic SNPs were used for association mapping. Association mapping was performed (Supplementary File 5) with *GAPIT 3.1.0* (Wang and Zhang 2021) within *R 4.2.1* (R2022) using GLM and BLINK models (Huang *et al*. 2019). The MLM (Yu *et al*. 2006), CMLM (Zhang *et al*. 2010), MLMM (Segura *et al*. 2012), and FarmCPU (Liu *et al*. 2016) models were also evaluated but showed sequential improvement of the model in between GLM and BLINK models and are therefore not reported. Genotyping files were recoded as “0” for homozygous reference allele calls, “1” for heterozygous calls, and “2” for homozygous alternative allele calls (Supplementary File 6). The genetic map file consisted of marker name, chromosome, and position (Supplementary File 7). The phenotyping file consisted of sample name and sex where “0” indicated males and “1” indicated females (Supplementary File 8). As BLINK makes use of linkage disequilibrium (LD) to form a kinship matrix, markers in LD were not removed and the kinship matrix was not calculated independently. In addition, accounting for the population structure using principal components did not lead to an improvement in the model and is therefore not reported here. The best model was selected based on a visual inspection of the QQ plot. The false discovery rate (FDR) and Bonferroni correction thresholds of 4.28 and 5.62 were calculated at the 0.05 confidence level, respectively. Haplotypes were constructed by assigning a value from 1 through 4 to each phased haplotype class (00, 01, 10, 11) for significant marker 1 (SM1, first digit) and significant marker 2 (SM2, second digit) and concatenated. For example, an accession with the phased genotypes 0|1 for SM1 and 0|1 for SM2 would become 00 and 11 with the passed haplotype of 1|4, whereas 0|1 for SM1 and 1|0 for SM2 would become 01 and 10 with a phased haplotype of 2|3. Candidate genes were identified by using nonsignificant neighboring markers to determine the physical interval underlying the genetic region using Geneious Prime 2022.2.1 (https://www.geneious.com). Coding sequences of candidate genes were extracted and translated to amino acid sequences within Geneious Prime 2022.2.1 and assessed using the Basic Local Alignment Search Tool (Sayers *et al*. 2022), UniProt (Coudert *et al*. 2023), InterProScan (Jones *et al*. 2014), and ProteInfer (Sanderson *et al*. 2023).

### Assay design, optimization, and validation

The single most significant SNP identified from the association mapping was used for assay development because of its high prediction accuracy rate of 96.2% for male and female hop plants. Competitive forward primers were designed manually in both the sense and the antisense orientation of the target SNP (Table 1) using Geneious Prime 2022.2.1. Oligonucleotides for both designs were ordered from Integrated DNA Technologies (Coralville, IA, USA) and combined with 12 µl of each forward primer, 30 µl of the common reverse primer, and 46 µl of water for working primer assay stock. PCRs were assessed on 5 male, 5 female, 2 monoecious plants, and an unknown, and 2 nontemplate control (NTC) samples were extracted using the Gentra Puregene DNA Extraction Kit (Qiagen, Venlo, The Netherlands). Reaction volumes of 15 µl containing 7.5 µl standard ROX PACE mastermix (3CR Bioscience, Harlow, UK) and primer assay, and 7.5 µl DNA in 96-well plate format on a CFX96 qPCR thermocycler (Bio-Rad Laboratories, Hercules, CA, USA). Cycle number was optimized by testing 26, 28, 30, 32, 34, and 36× cycles post touchdown of 10 cycles (61–55°C, −0.6°C/cycle). The best assay design was also determined during the cycling number optimization by assessing group clustering. Primer concentrations were optimized by adjusting the proportion of competitive forward allele primers but not the overall primer concentration using the optimal cycling number. All results were analyzed in CFX Manager 3.1 (Bio-Rad Laboratories).

**Table 1.** Competitive allele primers for the validated hop sex assay.

Primer	Sequence
Competitive forward 1	GAAGGTGACCAAGTTCATGCCCGTTCATGGATGGAAGC
Competitive forward 2	GAAGGTCGGAGTCAACGGATCCGTTCATGGATGGAAGA
Common reverse	TTCAACTCCCAGATGCCG

### DNA extraction optimization

Three DNA extraction protocols were tested to facilitate low cost and rapid turnaround from tissue sample collection to actionable data. The protocols consisted of HotSHOT (Truett *et al*. 2000), a custom HotSHOT extraction adapted to plants with high secondary metabolite concentrations (Fang *et al*. 1992; Xin *et al*. 2003; Noh *et al*. 2017), and QuickExtract (LGC, Biosearch Technologies, Hoddesdon, UK). Extractions were carried out on 3 identically formatted plates to assess the performance of the optimized assay. For all methods, ∼2–5 mm of plant tissue was collected, flash frozen, and lyophilized. For the HotSHOT method, 50 µl of alkaline lysis buffer (25 mM NaOH, 0.2 mM EDTA) was added and incubated at 95°C for 1 h. Subsequently, 50 µl of neutralization buffer (40 mM Tris-HCL) was added and pipette-mixed. For the modified HotSHOT method, 50 µl of alkaline lysis buffer (100 mM NaOH, 10% Tween 20) was added and incubated at 95°C for 10 min. Subsequently, 50 µl of neutralization buffer (100 mM Tris-HCL, 2 mM EDTA) was added, vortexed, and incubated at 4°C for at least 30 min. For the QuickExtract method, 50 µl of the QuickExtract solution was added to each well, vortexed, and incubated at 65°C for 15 min. Next, the samples were vortexed for 15 s before incubating at 95°C for 2 min to inactivate the solution.

### Assay validation and comparison with a recent diagnostic marker

Following assay and DNA extraction optimization, a total of 94 samples consisting of 64 females and 30 males from 15 independent populations were used to validate the diagnostic marker (Supplementary File 2). DNA was extracted from fresh tissue using the modified HotSHOT method at Prosser, WA. Samples were shipped on ice within 7 days from extraction to the NCGR in Corvallis, OR and diluted 50×- to 100×-fold using distilled water. The assay was performed in 10 µl reactions using the developed primer assay determined during assay optimization. An additional 299 samples consisting of 238 females and 61 males were used for comparison to a recently developed marker (Supplementary File 2; Havill *et al*. 2023). DNA was extracted by LGC (Biosearch Technologies) and shipped to NCGR in Corvallis, OR and reaction conditions remained the same as the previous validation set.

## Results

### Sequencing and data curation

The mean raw sequence read count was 5.4 million per sample for the initial 1,152 samples. A total of 12.7 million (12,705,880) variants were called before filtering. Post filtering, a total of 250 samples were removed due to an inadequate amount of data along with the 22 blank controls. A total of 90 replications of accessions samples were removed, as well as a further 25 samples were removed due to the inability to obtain accurate phenotypic information. Therefore, a total of 765 samples (Supplementary File 1) and 20,861 biallelic SNPs were used for association mapping.

### Association mapping and candidate gene assessment

**Fig. 1.**
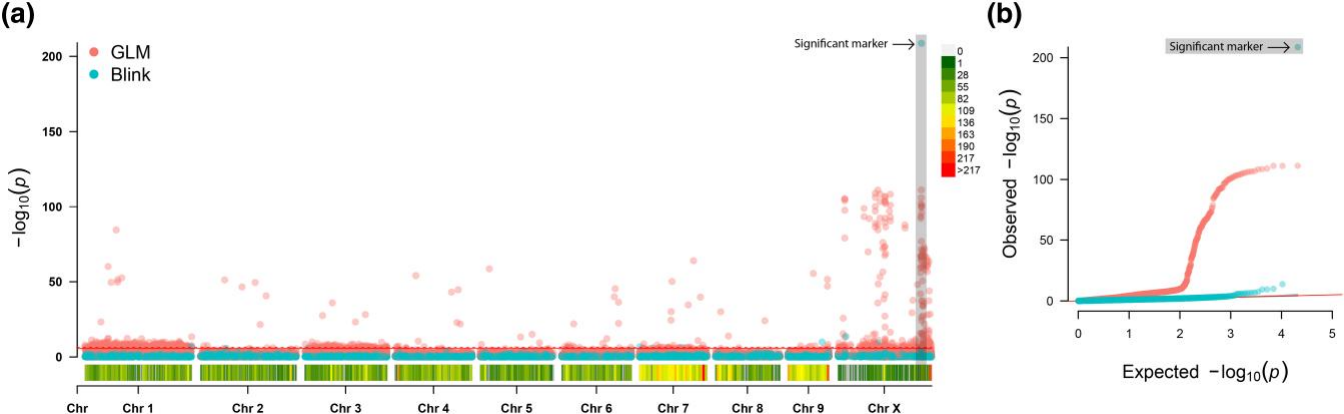
a) Manhattan plot of the association models for hop sex using GLM and BLINK algorithms. Chromosomes and marker density are indicated on the *x*-axis, where marker densities are plotted as the number of markers per 10 Mb window, with low and high marker density as represented by heat map on the right. The most significant marker used for marker development is highlighted with a vertical bar. The LOD score is on the *y*-axis with Bonferroni thresholds indicated by the solid (*α*-level 0.05) and dashed (*α*-level 0.01) lines. b) Quantile–quantile plot for the GLM and BLINK association models for hop sex with the expected LOD score on the *x*-axis and observed LOD scores on the *y*-axis. The solid line indicates whether all data points did not deviate from the expected LOD score.

**Fig. 2.**
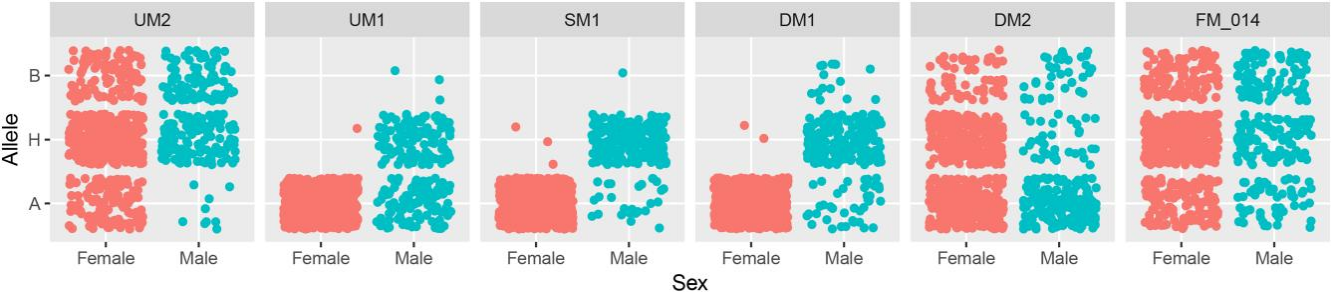
Jitter plot of allelic distribution of markers surrounding the significant marker from the association mapping. Two flanking markers upstream (UM2 and UM1) and 2 markers downstream (DM1 and DM2) of the most SM1 are shown in physical order from the association mapping. Each panel is separated into female (left) and male (right) and allele class of homozygous reference (A), heterozygous (H), and homozygous alternative (B).

The GLM model identified a total of 1,984 and 1,143 significant markers distributed across all 10 chromosomes above the FDR and Bonferroni correction thresholds, respectively. A total of 353 (17.8%) and 455 (39.8%) were identified on the hop sex chromosome X using the FDR and Bonferroni correction thresholds, respectively. However, the BLINK model had a total of 19 and 16 significant markers above the FDR and Bonferroni correction thresholds, respectively. Of those, 7 (36.8%) and 7 (43.8%) were identified on the hop sex chromosome. However, a single marker with a LOD score of 208.7 was identified on the sex chromosome of hop, whereas the second most significant marker had a LOD score of 13.7. This single marker was 96.2% (736/765) accurate in prediction of male or female hop plants in the association mapping panel and further used as the target SNP for assay design (Fig. 1). Two markers upstream (UM1 and UM2) and downstream (DM1 and DM2) of significant marker 1 (SM1) were used to determine if any additional SNPs in close proximity could also be targeted for assay design (Fig. 2). However, predictive ability rapidly declined in both directions from the identified SNP. In addition, attempts to assign haplotypes using flanking markers or SM2 failed to significantly boost predictive accuracy (Supplementary Figure 1). The 2 nonsignificant neighboring markers delimit a 139 kb physical interval with a total of 8 genes within the “Cascade” reference genome (Padgitt-Cobb *et al*. 2023). Only one gene (000120F.g41) is predicted to encode a protein with function as a basic helix-loop-helix (bHLH) transcription factor, whereas the remaining genes are hypothetical proteins.

### Assay design, optimization, and validation

**Fig. 3.**
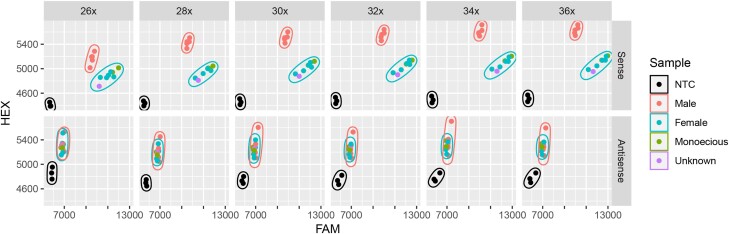
Scatter plot of DNA assay for design assay design and cycling conditions. Fluorescence from the FAM fluorophore is on the *x*-axis and HEX fluorophore is on the *y*-axis, with the sense design on the top facet and antisense design on the bottom facet. Number of standard PCR cycles posttouchdown ranging from 26× to 36× cycles are shown in 2× increments for both sense and antisense designs. NTCs, males, females, monoecious plants and unknown samples are shown. Clusters are outlined as NTCs, male, and female.

Assay designs with competitive forward primers in both the sense and antisense directions were tested, where sense primers resulted in clearly defined clusters for both males and females compared with the antisense design (Fig. 3). The cycling number of 30× was chosen for future reactions, as sample cluster migration had plateaued; however, the plate was not overcycled to the point where NTCs began to migrate. The sense design was therefore carried forward for further optimization and evaluation. Adjusting primer concentrations to boost either the FAM- or HEX-specific allele did not significantly improve or detract from clustering of samples and therefore remained at standard levels (Supplementary Figure 2).

### DNA extraction optimization

**Fig. 4.**
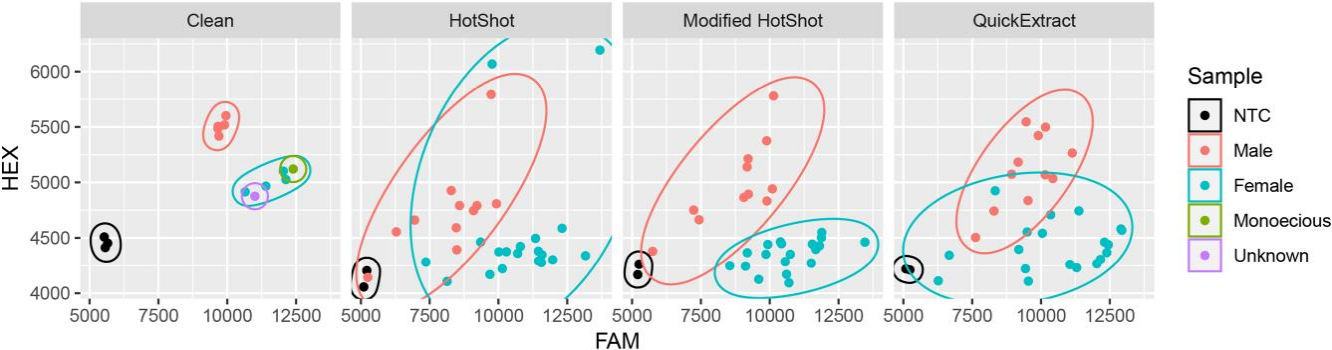
Scatter plot of DNA assay using different DNA extraction methods. Fluorescence from the FAM fluorophore is on the *x*-axis and HEX fluorophore is on the *y*-axis. Crude DNA extraction methods include HotSHOT, modified HotSHOT, and QuickExtract extraction protocols along with “clean” DNA from Gentra Puregene (30× sense panel of Fig. 3) are shown for comparison. NTCs, males, females, monoecious, and unknown samples are shown. Clusters are outlined as NTC, male, and female.

DNA extraction remained a major financial burden for obtaining actionable data due to the cost of kits and disposable consumables. Therefore, multiple rapid DNA extraction tests were trialed to determine if clean data point clustering could still be achieved without “clean” DNA. The 50–100× dilution using the modified HotSHOT DNA extraction method (Fang *et al*. 1992; Xin *et al*. 2003; Noh *et al*. 2017) provided separated clusters, whereas the standard HotSHOT and QuickExtract showed cluster overlap (Fig. 4).

### Assay validation

**Fig. 5.**
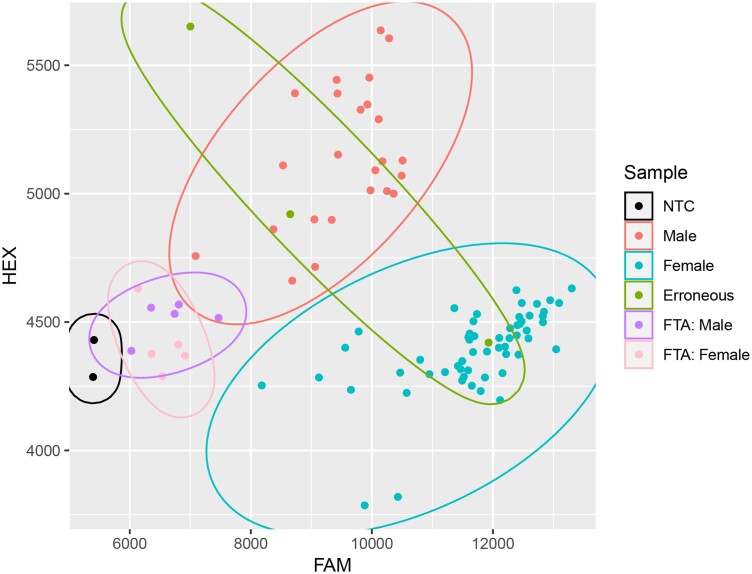
Scatter plot of the DNA assay using the validation plate. Fluorescence from the FAM fluorophore is on the *x*-axis and HEX fluorophore on the *y*-axis utilizing the modified HotSHOT and optimized assay design. NTCs, males, females, erroneous samples, failed to amplify (FTA) male and female, respectively, are shown. Clusters are outlined as NTC, male, female, FTA: male, and FTA: female.

**Fig. 6.**
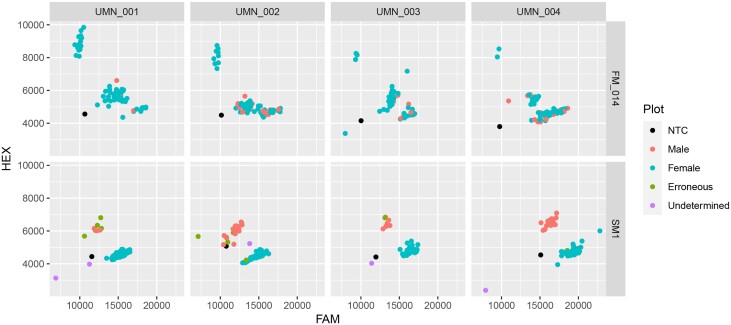
Scatter plot of DNA assay in comparison with a recently developed assay (Havill *et al*. 2023). Fluorescence from the FAM fluorophore on the *x*-axis and HEX fluorophore on the *y*-axis with the previously developed assay (FM014) on the top facet and the DNA assay from this study on the bottom facet using 4 validation plates from the University of Minnesota (UMN). NTCs, males, females, erroneous, and undetermined samples are shown.

Overall, both male and female samples cluster in defined groups, and NTCs remain unamplified suggesting that contamination was not present (Fig. 5). SM1 correctly identified 57/64 females and 24/30 males on the validation plate. However, 5 females and 5 males are accounted for by the samples failing to amplify and not included in the accuracy calculation. The remaining two female samples and one male sample were clustered incorrectly. Therefore, the marker has an accuracy of 96% correctly identifying 80/83 samples that were amplified. Further investigation revealed that one of these samples and an adjacent sample that failed to amplify may have been inadvertently mixed up from sampling error in the field. We sought to compare our marker with a recently developed marker (FM_014; Havill *et al*. 2023) that is in close proximity to SM1 on validation material previously used for FM_014 validation. FM_014 was screened on 299 accessions consisting of 238 females and 61 males, and can accurately cluster females (homozygous HEX) with up to 2 additional clusters (heterozygotes and homozygous FAM) being a mix of males and females, making accuracy calculations and determining erroneous calls difficult (Fig. 6). However, SM1 generates 2 clusters that are female (homozygous FAM) and male (heterozygous), respectively, with 94% accuracy and 275 correctly identified (5 undetermined) accessions using the set of 299 accessions (Fig. 6). A total of 7 out of the 19 erroneous calls with SM1 are in *H. lupulus* var. *neomexicanus* and var. *pubescens* (Supplementary File 2).

## Discussion

Using only the GLM method of GAPIT, a total of 1,984 and 1,143 markers across all 10 chromosomes were identified above the 0.05 FDR threshold and Bonferroni correction thresholds, respectively. Considering that *H. lupulus* uses an XY sex determination system (Parker and Clark 1991; Shephard *et al*. 2000), this appears highly inflated with substantial false positives throughout the genome. Adoption of modern association mapping algorithms such as BLINK (Huang *et al*. 2019), which typically reduce the number of false-positive associations, while simultaneously inflating potentially true associations, allowed the identification of significantly associated markers. The most significant marker (1533_377039504, SM1) had a LOD score of 208.7, whereas the second most significant marker (1533_40241856, SM2) was substantially lower with a LOD score of 13.7. Despite additional significant markers, SM1 had a prediction accuracy of 96.2%, whereas the next most significant marker had a prediction accuracy of 51.5%. When SM1 and SM2 were combined into a haplotype block this failed to identify any additional accessions into the correctly assigned group. This can be seen where the majority of female accessions are 1|1, 1|2, 2|1, or 2|2, whereas the majority of males are the remaining haplotype classes (predominantly 1|3 or 3|1; Supplementary Figure 1). Therefore, SM2 did not warrant marker development. There is also the possibility that the remaining 0.8% of samples that were incorrectly identified may have been due to phenotyping error, sampling error, sequencing error, or other unknown sources of error. This was investigated further in the 2023 field season: 18 of the 27 accessions where the marker did not predict hop plant sex are now in concordance with SM1 while 7 of the 765 accessions originally considered in concordance with SM1 are now not in concordance. This results in 748 out of 765 hop plants being accurately predicted using SM1 with an updated prediction accuracy of 97.8% (Supplementary Figure 3 and File 8).

The female genome assembly of “Cascade” recently identified Scaffold_1533 as the sex chromosome and renamed it as chromosome X (Padgitt-Cobb *et al*. 2023). The fact that SM1 is located within a 139 kb physical interval on chromosome X with a LOD score of 208.7 provides confidence that this marker is linked to hop sex. In addition, the fact that the marker is heterozygous in males and homozygous in females suggests this region is within the sex-specific (nonrecombining) region of the sex chromosome, and the male-specific allele is linked to chromosome Y, despite this region previously being linked to the pseudoautosomal (recombining) region of the X/Y chromosomes (Padgitt-Cobb *et al*. 2021). Lastly, the SM1 diagnostic marker is co-dominant (not dominant presence/absence variation), amplifying in males and females and therefore, there is no ambiguity as to whether specific samples fail to amplify the target SNP. Recently, a 47.5 Mb region was mapped within a Zenith x 21058M biparental population (Havill *et al*. 2023), spanning the 139 kb physical interval mapped in this study. The peak marker (FM_014) identified in the Zenith x 21058M population is located 757.5 kb downstream of SM1, within a RING-type E3 ubiquitin transferase gene. This peak marker exhibited higher transferability than 3 additional markers that were converted to diagnostic assays in the Zenith population study (Havill *et al*. 2023). However, FM_014 was also present within the present association mapping study (1533_377797048) with a LOD score of 0.5, suggesting FM_014 marker is not in close linkage in a wider diversity of germplasm. The 139 kb physical interval delimited within the present study contains 8 genes, only one of which is predicted to have homology to a functional protein. This single gene is predicted to encode a bHLH transcription factor protein. Genes within the bHLH family have known functions in male sterility, such as *DYT1* and *AMS* in *Arabidopsis* (Sorensen *et al*. 2003; Zhang *et al*. 2006; Xu *et al*. 2014), *UDT1* and TDR in rice (Jung *et al*. 2005; Li *et al*. 2006), *MS32* in maize (Nan *et al*. 2017), *MS10* in tomato (Jeong *et al*. 2014), *ATM1* in watermelon (Zhang *et al*. 2021), and *BNB* in liverwort (Yamaoka *et al*. 2018; Hisanaga *et al*. 2019). The SM1 marker results in a M78I amino acid substitution that may therefore influence binding affinity to male-specific genes to facilitate male hop development.

A total of 112 of 265 genes were identified as homologous and sex linked between hops and the closely related species *Cannabis sativa* (Prentout *et al*. 2021). However, these sex-linked genes were identified within the sex-specific region of the chromosome (Prentout *et al*. 2021), making a co-dominant diagnostic marker development troublesome. Recent competitive allele-specific primers developed have been evaluated on 2,170 plants, but only represented 14 hemp cultivars (Toth *et al*. 2020) and may therefore not transfer to a wider diversity of *C. sativa* accessions. Despite the close phylogenetic relationship of *H. lupulus* and *C. sativa*, the *C. sativa* primer sequences were not identified in the “Cascade” hop genome assembly, and therefore are not expected to transfer to hop. In addition, in the 299-validation set, the SM1 marker failed to predict 19/299 accessions accurately for female or male phenotype, which corresponded to wild North American botanical varieties of hops such as var. *neomexicanus* and var. *pubescens* (Supplementary File 2), further supporting the expected lack of transferability to the more distantly related *C. sativa*. With high-resolution mapping delimiting 8 genes, only one of which is predicted to function in male sterility and contains SM1, there is high confidence that if the marker is not causal, then it has extremely tight linkage to hop sex determination and will ultimately be transferable to additional breeding programs or uncharacterized European hop germplasm. The fact that the antisense assay design failed to distinguish sex in the test accessions suggests there may be additional mutations within close proximity that may affect primer binding.

A current limitation to the identification of universally perfect sex-linked markers in hop is the lack of a publicly available and highly contiguous Y chromosome assembly. In dioecious plant and animal species, bias in the sex of the reference genome used for read mapping complicates most downstream analyses that involve multiple sexes, given that sex chromosomes can vary greatly from each other and from autosomes (Carey *et al*. 2022). One constraint in this study is that all sequencing data, regardless of sex, were aligned to an XX female “Cascade” reference genome, meaning that Y-specific reads in males are likely unmappable, and association mapping is limited mostly to sequences that share homology between X and Y. Building fully phased genome representations of both X and Y chromosomes for diverse genotypes would help to better distinguish Y-specific or highly diverged regions from the homologous regions that are better suited to co-dominant marker development. In addition, a Y reference would identify Y-specific SNPs that may impact primer binding. With recent advances in long-read genome sequencing and assembly, as well as lower costs, assembling XY pairs in a complex species with a large genome like hops is now feasible (Carey *et al*. 2021; Cauret *et al*. 2022; Zhang *et al*. 2022; Yue *et al*. 2023). Coupling phased nuclear assemblies with low-coverage whole-genome sequencing across diverse germplasm is poised to reveal the standing nucleotide and structural diversity of sex chromosomes and will certainly contribute to the improvement of universal sex-linked markers over time.

Currently, the SM1 diagnostic marker is unable to distinguish monoecious plants from female hop plants (Figs. 3 and 5, Supplementary Figure 2). In theory, monoecious plants are hypothesized to occur due to deviating from the 0.5 to 1.0 allelic balance (Parker and Clark 1991; Shephard *et al*. 2000) and should therefore cluster between male and female groups. However, this is not currently the case with the SM1 diagnostic marker. Limited research has shown that auxin has a masculinizing effect on hop plants, resulting in male inflorescence on female hop plants (Heslop-Harrison 1963). Auxin is known to be important in the adaptive response of plants to drought stress (Verma *et al*. 2022), which is further corroborated by anecdotal evidence from the increase in the monoecious plants documented in Washington during the 2022 growing season which experienced higher than average temperatures during flowering. Therefore, additional association mapping could be conducted on stressed female hop plants to identify which female plants have the potential to result in monoecious plants through inducible stress responses.

Numerous breeding programs may take up to 2 years to determine whether newly developed hop seedlings are female or male (Prentout *et al*. 2021), thus wasting valuable time that could be repurposed either for additional female evaluation or for cost reductions by not maintaining numerous male hop plants. A 4-marker SSR multiplex has been used in Slovenian breeding programs (Čerenak *et al*. 2019) to screen out males, but it is limited by its low-throughput, long assay time, and requirement of human interpretation of the results. In comparison, using this validated SNP assay, data can be easily obtained under 4 h from the time of tissue collection using a modified HotSHOT DNA extraction (Fang *et al*. 1992; Xin *et al*. 2003; Noh *et al*. 2017) and marker assay that can be performed on any qPCR instrument. Furthermore, our cost analysis predicts that actionable data can be achieved at a cost of $0.15 per data point using a 96-well DNA extraction and 384-well marker assay format. This is equivalent to $150 per 1,000 samples, which substantially lowers the financial burden and time constraints, while simultaneously increasing sample throughput. This marker has the potential to rapidly cut 1–2 years out of cultivar release timelines, allowing a reallocation of resources to additional areas of crop improvement such as resilience to climate change, disease resistance, agronomic traits, or consumer trends.

## Conclusions

Breeding programs are currently limited by the time-consuming wait to determine whether hop plants are male or female using visual observations. This hop sex determination marker assay combines a rapid in-house DNA extraction and marker assay utilizing low-cost reagents, as well as equipment and instruments typically found in molecular laboratories. Therefore, hop breeding programs can increase their efficiency (time, labor, money, and space) to release cultivars earlier, as well as increase their genetic gain to counter pressures such as climate change and disease and address changing consumer trends, by reallocating resources to other aspects of the hop breeding program.

## Data Availability

Raw sequencing data generated were submitted to the NCBI Sequence Read Archive database and are openly available under BioProject ID PRJNA1004990 (https://www.ncbi.nlm.nih.gov/bioproject/1004990) and BioSamples accession numbers SAMN36969246 through SAMN36970010. Data files and software code for data curation and association analysis have been submitted to figshare: https://doi.org/10.25387/g3.23938038. Supplementary File 1 contains accession information used for association mapping. Supplementary File 2 contains accession information used for validation. Supplementary File 3 contains the variant calling script. Supplementary File 4 contains the filtered and imputed vcf. Supplementary File 5 contains the association mapping and figure construction script. Supplementary File 6 contains genotyping data. Supplementary File 7 contains the genetic map. Supplementary File 8 contains phenotyping data.

## References

[jkad216-B1] Andrews S . 2010. FastQC: A Quality Control Tool for High Throughput Sequence Data. [Online]. http://www.bioinformatics.babraham.ac.uk/projects/fastqc/.

[jkad216-B2] Buck EJ, Wiedow C, Carlisle C, Chagné D, Beatson R. 2009. Validation of new sex specific DNA markers for hops (*Humulus lupulus* L.). Acta Hortic. 848:323–328. doi:10.17660/ActaHortic.2009.848.33.

[jkad216-B4] Carey S, Yu Q, Harkess A. 2021. The diversity of plant sex chromosomes highlighted through advances in genome sequencing. Genes (Basel). 12(3):381. doi:10.3390/genes12030381. 33800038 PMC8000587

[jkad216-B3] Carey SB, Lovell JT, Jenkins J, Leebens-Mack J, Schmutz J, Wilson MA, Harkess A. 2022. Representing sex chromosomes in genome assemblies. Cell Genom. 2(5):100132. doi:10.1016/j.xgen.2022.100132. 35720975 PMC9205529

[jkad216-B5] Cauret CM, Mortimer SM, Roberti MC, Ashman TL, Liston A. 2022. Chromosome-scale assembly with a phased sex-determining region resolves features of early Z and W chromosome differentiation in a wild octoploid strawberry. G3. 12 (8):jkac139. doi:10.1093/g3journal/jkac139. PMC9339316 35666193

[jkad216-B6] Čerenak A, Kolenc Z, Sehur P, Whittock SP, Koutoulis A, Beatson R, Buck E, Javornik B, Škof S, Jakše J. 2019. New male specific markers for hop and application in breeding program. Sci Rep. 9(1):14223. doi:10.1038/s41598-019-50400-z. 31578340 PMC6775077

[jkad216-B7] Čerenak A, Satovic Z, Jakse J, Luthar Z, Carovic-Stanko K, Javornik B. 2009. Identification of QTLs for alpha acid content and yield in hop (*Humulus lupulus* L.). Euphytica. 170(1–2):141–154. doi:10.1007/s10681-009-9920-9.

[jkad216-B8] Čerenak A, Satovic Z, Javornik B. 2006. Genetic mapping of hop (*Humulus lupulus* L.) applied to the detection of QTLs for alpha-acid content. Genome. 49(5):485–494. doi:10.1139/g06-007. 16767173

[jkad216-B9] Chen S, Zhou Y, Chen Y, Gu J. 2018. fastp: an ultra-fast all-in-one FASTQ preprocessor. Bioinformatics. 34(17):i884–i890. doi:10.1093/bioinformatics/bty560. 30423086 PMC6129281

[jkad216-B10] Coudert E, Gehant S, de Castro E, Pozzato M, Baratin D, Neto T, Sigrist CJA, Redaschi N, Bridge A, Bridge AJ, et al 2023. Annotation of biologically relevant ligands in UniProtKB using ChEBI. Bioinformatics. 39(1):btac793. doi:10.1093/bioinformatics/btac793. 36484697 PMC9825770

[jkad216-B112] Danecek P, Auton A, Abecasis G, Albers CA, Banks E, DePristo MA, Handsaker RE, Lunter G, Marth GT, Sherry ST, et al 2011. The variant call format and VCFtools. Bioinformatics. 27(15):2156–2158. doi:10.1093/bioinformatics/btr330. 21653522 PMC3137218

[jkad216-B111] Danilova TV, Karlov GI. 2006. Application of inter simple sequence repeat (ISSR) polymorphism for detection of sex-specific molecular markers in hop (Humulus lupulus L.). Euphytica. 151(1):15–21. doi:10.1093/bioinformatics/btr330.

[jkad216-B12] Divashuk MG, Alexandrov OS, Kroupin PY, Karlov GI. 2011. Molecular cytogenetic mapping of *Humulus lupulus* sex chromosomes. Cytogenet Genome Res. 134:213–219. doi:10.1159/000328831. 21709414

[jkad216-B13] Donner P, Pokorný J, Ježek J, Krofta K, Patzak J, Pulkrábek J. 2020. Influence of weather conditions, irrigation and plant age on yield and alpha-acids content of Czech hop (*Humulus lupulus* L.) cultivars. Plant Soil Environ. 66(1):41–46. doi:10.17221/627/2019-PSE.

[jkad216-B14] Driskill M, Pardee K, Hummer KE, Zurn JD, Amundsen K, Wiles A, Wiedow C, Patzak J, Henning JA, Bassil NV. 2022. Two fingerprinting sets for *Humulus lupulus* based on KASP and microsatellite markers. PLoS One. 17(4):e0257746. doi:10.1371/journal.pone.0257746. 35421090 PMC9009645

[jkad216-B114] Elshire RJ, Glaubitz JC, Sun Q, Poland JA, Kawamoto K, Buckler ES, Mitchell SE. 2011. A robust, simple genotyping-by-sequencing (GBS) approach for high diversity species. PloS one. 6(5):e19379. doi:10.1371/journal.pone.0019379. 21573248 PMC3087801

[jkad216-B15] Ewels P, Magnusson M, Lundin S, Käller M. 2016. MultiQC: summarize analysis results for multiple tools and samples in a single report. Bioinformatics. 32(19):3047–3048. doi:10.1093/bioinformatics/btw354. 27312411 PMC5039924

[jkad216-B16] Fang G, Hammar S, Grumet R. 1992. A quick and inexpensive method for removing polysaccharides from plant genomic DNA. Biotechniques. 13(1):52–54, 56. https://europepmc.org/article/med/1503775. 1503775

[jkad216-B17] Haunold A . 1981. Hop production, breeding, and variety development in various countries. J Am Soc Brew Chem. 39(1):27–34. doi:10.1094/ASBCJ-39-0027.

[jkad216-B117] Haunold A, Zimmermann CE. 1974. Pollen collection, crossing, and seed germination of hop. Crop Science. 14(5):774–776. doi:10.2135/cropsci1974.0011183X001400050051x.

[jkad216-B18] Havill JS, Richardson BJ, Rohwer CL, Gent DH, Henning JA, Muehlbauer GJ. 2023. Identification of quantitative trait loci associated with *R1*-mediated resistance to powdery mildew and sex determination in hop (*Humulus lupulus* L.). Theor Appl Genet. 136(7):1534. doi:10.1007/s00122-023-04399-7. 37318664

[jkad216-B19] Henning J, Gent DH, Townsend MS, Woods JL, Hill ST, Hendrix D. 2017. QTL analysis of resistance to powdery mildew in hop (*Humulus lupulus* L.). Euphytica. 213(4):98. doi:10.1007/s10681-017-1849-9.

[jkad216-B21] Henning J, Hill S, Darby P, Hendrix D. 2017. QTL examination of a bi-parental mapping population segregating for “short-stature” in hop (*Humulus lupulus* L.). Euphytica. 213(3):77. doi:10.1007/s10681-017-1848-x.

[jkad216-B20] Henning JA, Gent DH, Twomey MC, Townsend MS, Pitra NJ, Matthews PD. 2015. Precision QTL mapping of downy mildew resistance in hop (*Humulus lupulus* L.). Euphytica. 202(3):487–498. doi:10.1007/s10681-015-1356-9.

[jkad216-B22] Henning JA, Townsend MS, Gent DH, Bassil N, Matthews P, Buck E, Beatson R. 2011. QTL mapping of powdery mildew susceptibility in hop (*Humulus lupulus* L.). Euphytica. 180(3):411–420. doi:10.1007/s10681-011-0403-4.

[jkad216-B23] Heslop-Harrison J . 1963. Ultrastructural aspects of differentiation in sporogenous tissue. Symp Soc Exp Biol. 17:315–340. https://pubmed.ncbi.nlm.nih.gov/5849043/5849043

[jkad216-B24] Hill ST, Sudarsanam R, Henning J, Hendrix D. 2017. HopBase: a unified resource for Humulus genomics. Database. 2017(1):bax009. doi:10.1093/database/bax009. 28415075 PMC5467566

[jkad216-B25] Hisanaga T, Yamaoka S, Kawashima T, Higo A, Nakajima K, Araki T, Kohchi T, Berger F. 2019. Building new insights in plant gametogenesis from an evolutionary perspective. Nat Plants. 5(7):663–669. doi:10.1038/s41477-019-0466-0. 31285561

[jkad216-B26] Huang M, Liu X, Zhou Y, Summers RM, Zhang Z. 2019. BLINK: a package for the next level of genome-wide association studies with both individuals and markers in the millions. GigaScience. 8(2):1–12. doi:10.1093/gigascience/giy154. PMC6365300 30535326

[jkad216-B27] Jakse J, Stajner N, Kozjak P, Cerenak A, Javornik B. 2008. Trinucleotide microsatellite repeat is tightly linked to male sex in hop (*Humulus lupulus* L.). Mol Breed. 21(2):139–148. doi:10.1007/s11032-007-9114-x.

[jkad216-B28] Jeong H-J, Kang J-H, Zhao M, Kwon J-K, Choi H-S, Bae JH, Lee H-A, Joung Y-H, Choi D, Kang B-C. 2014. Tomato *Male sterile* 10^35^ is essential for pollen development and meiosis in anthers. J Exp Bot. 65(22):6693–6709. doi:10.1093/jxb/eru389. 25262227 PMC4246194

[jkad216-B29] Jones P, Binns D, Chang H-Y, Fraser M, Li W, McAnulla C, McWilliam H, Maslen J, Mitchell A, Nuka G, et al 2014. InterProScan 5: genome-scale protein function classification. Bioinformatics. 30(9):1236–1240. doi:10.1093/bioinformatics/btu031. 24451626 PMC3998142

[jkad216-B30] Jung K-H, Han M-J, Lee Y-S, Kim Y-W, Hwang I, Kim M-J, Kim Y-K, Nahm BH, An G. 2005. Rice *Undeveloped Tapetum1* is a major regulator of early tapetum development. Plant Cell. 17(10):2705–2722. doi:10.1105/tpc.105.034090. 16141453 PMC1242267

[jkad216-B31] Koie K, Inaba A, Okada Y, Kaneko T, Ito K. 2005. Construction of the genetic linkage map and QTL analysis on hop (*Humulus lupulus* L.). Acta Hortic. 668:59–66. doi:10.17660/ActaHortic.2005.668.7.

[jkad216-B32] Lafontaine S, Varnum S, Roland A, Delpech S, Dagan L, Vollmer D, Kishimoto T, Shellhammer T. 2019. Impact of harvest maturity on the aroma characteristics and chemistry of Cascade hops used for dry-hopping. Food Chem. 278:228–239. doi:10.1016/j.foodchem.2018.10.148. 30583367

[jkad216-B33] Li H, Handsaker B, Wysoker A, Fennell T, Ruan J, Homer N, Marth G, Abecasis G, Durbin R. 2009. The Sequence Alignment/Map format and SAMtools. Bioinformatics. 25(16):2078–2079. doi:10.1093/bioinformatics/btp352. 19505943 PMC2723002

[jkad216-B34] Li N, Zhang D-S, Liu H-S, Yin C-S, Li X, Liang W-Q, Yuan Z, Xu B, Chu H-W, Wang J, et al 2006. The rice *Tapetum Degeneration Retardation* gene is required for tapetum degradation and anther development. Plant Cell. 18(11):2999–3014. doi:10.1105/tpc.106.044107. 17138695 PMC1693939

[jkad216-B35] Liu X, Huang M, Fan B, Buckler ES, Zhang Z. 2016. Iterative usage of fixed and random effect models for powerful and efficient genome-wide association studies. PLoS Genet. 12(2):e1005767. doi:10.1371/journal.pgen.1005767. 26828793 PMC4734661

[jkad216-B36] McAdam EL, Freeman JS, Whittock SP, Buck EJ, Jakse J, Cerenak A, Javornik B, Kilian A, Wang C-H, Andersen D, et al 2013. Quantitative trait loci in hop (*Humulus lupulus* L.) reveal complex genetic architecture underlying variation in sex, yield and cone chemistry. BMC Genomics. 14(1):360. doi:10.1186/1471-2164-14-360. 23718194 PMC3680207

[jkad216-B37] McCallum JL, Nabuurs MH, Gallant ST, Kirby CW, Mills AAS. 2019. Phytochemical characterization of wild hops (*Humulus lupulus* ssp. *lupuloides*) germplasm resources from the Maritimes Region of Canada. Front Plant Sci. 10:1438. doi:10.3389/fpls.2019.01438. 31921222 PMC6917649

[jkad216-B38] Nan G-L, Zhai J, Arikit S, Morrow D, Fernandes J, Mai L, Nguyen N, Meyers BC, Walbot V. 2017. MS23, a master basic helix-loop-helix factor, regulates the specification and development of the tapetum in maize. Development. 144(1):163–172. doi:10.1242/dev.140673. 27913638

[jkad216-B39] USDA NASS . 2022. National hop report. USDA Annual Reports. [accessed 2022 Dec 19]. usahops.org.

[jkad216-B40] Noh Y-H, Lee S, Whitaker VM, Cearley KR, Cha J-S. 2017. A high-throughput marker-assisted selection system combining rapid DNA extraction high-resolution melting and simple sequence repeat analysis: strawberry as a model for fruit crops. J Berry Res. 7(1):23–31. doi:10.3233/JBR-160145.

[jkad216-B41] Olatoye MO, Wiseman M, Gent DH, Henning JA, Altendorf KR. 2023. Genetic characterization of downy mildew resistance from the hop (*Humulus lupulus* L.) line USDA 64035M. Crop Sci. 63(3):1082–1091. doi:10.1002/csc2.20880.

[jkad216-B42] Padgitt-Cobb LK, Kingan SB, Henning JA. 2019. Genomic analysis of powdery mildew resistance in a hop (*Humulus lupulus* L.) bi-parental population segregating for “R6-locus”. Euphytica. 216(1):10. doi:10.1007/s10681-019-2543-x.

[jkad216-B43] Padgitt-Cobb LK, Kothen-Hill S, Henning J, Hendrix J. 2021. The long-read genome assembly of hop (*Humulus lupulus*) uncovers the pseudoautosomal region and other genomic features. Acta Hortic. 1328(1328):1–16. doi:10.17660/ActaHortic.2021.1328.1.

[jkad216-B44] Padgitt-Cobb LK, Pitra NJ, Matthews PD, Henning JA, Hendrix DA. 2023. An improved assembly of the “Cascade” hop (*Humulus lupulus*) genome uncovers signatures of molecular evolution and refines time of divergence estimates for the Cannabaceae family. Hortic Res. 10(2):uhac281. doi:10.1093/hr/uhac281. 36818366 PMC9930403

[jkad216-B45] Paguet AS, Siah A, Lefèvre G, Sahpaz S, Rivière C. 2022. Agronomic, genetic and chemical tools for hop cultivation and breeding. Phytochem Rev. 21(2):667–708. doi:10.1007/s11101-022-09813-4.

[jkad216-B46] Parker JS, Clark MS. 1991. Dosage sex-chromosome systems in plants. Plant Sci. 80(1–2):79–92. doi:10.1016/0168-9452(91)90274-C.

[jkad216-B47] Patzak J, Henychova A, Kro K. 2012. Study of molecular markers for Xanthohumol and DMX contents in hop (*Humulus lupulus* L.) by QTLs mapping analysis. Brew Sci. 65:96–102.

[jkad216-B48] Polley A, Ganal MW, Seigner E. 1997. Identification of sex in hop (*Humulus lupulus*) using molecular markers. Genome. 40(3):357–361. doi:10.1139/g97-048. 18464833

[jkad216-B49] Pook T, Mayer M, Geibel J, Weigend S, Cavero D, Schoen CC, Simianer H. 2020. Improving imputation quality in BEAGLE for crop and livestock data. G3 (Bethesda). 10:177–188. doi:10.1534/g3.119.400798. 31676508 PMC6945036

[jkad216-B50] Poplin R, Ruano-Rubio V, DePristo MA, Fennell TJ, Carneiro MO, Van der Auwera GA, Kling DE, Gauthier LD, Levy-Moonshine A, Roazen D, et al 2018. Scaling accurate genetic variant discovery to tens of thousands of samples. bioRxiv 201178. 10.1101/201178, preprint: not peer reviewed.

[jkad216-B51] Prentout D, Stajner N, Cerenak A, Tricou T, Brochier-Armanet C, Jakse J, Käfer J, Marais GAB. 2021. Plant genera *Cannabis* and *Humulus* share the same pair of well-differentiated sex chromosomes. New Phytol. 231:1599–1611. doi:10.1111/nph.17456. 33978992

[jkad216-B52] Rasheed A, Hao Y, Xia X, Khan A, Xu Y, Varshney RK, He Z. 2017. Crop breeding chips and genotyping platforms: progress, challenges, and perspectives. Mol Plant. 10:1047–1064. doi:10.1016/j.molp.2017.06.008. 28669791

[jkad216-B53] R Core Team . 2022. R: A Language and Environment for Statistical Computing. Vienna: R Foundation for Statistical Computing. https://www.R-project.org/.

[jkad216-B54] Roehr JT, Dieterich C, Reinert K. 2017. Flexbar 3.0—SIMD and multicore parallelization. Bioinformatics. 33:2941–2942. doi:10.1093/bioinformatics/btx330. 28541403

[jkad216-B55] Sanderson T, Bileschi ML, Belanger D, Colwell LJ. 2023. ProteInfer, deep neural networks for protein functional inference. eLife. 12:e80942. doi:10.7554/eLife.80942. 36847334 PMC10063232

[jkad216-B56] Sayers EW, Bolton EE, Brister JR, Canese K, Chan J, Comeau DC, Connor R, Funk K, Kelly C, Kim S, et al 2022. Database resources of the national center for biotechnology information. Nucleic Acids Res. 50:D20–D26. doi:10.1093/nar/gkab1112. 34850941 PMC8728269

[jkad216-B57] Segura V, Vilhjálmsson BJ, Platt A, Korte A, Seren Ü, Long Q, Nordborg M. 2012. An efficient multi-locus mixed-model approach for genome-wide association studies in structured populations. Nat Genet. 44:825–830. doi:10.1038/ng.2314. 22706313 PMC3386481

[jkad216-B58] Seigner E, Lutz A, Oberhollenzer K, Seidenberger R, Seefelder S, Felsenstein F. 2009. Breeding of hop varieties for the future. Acta Hortic. 49–58. doi:10.17660/ActaHortic.2009.848.4.

[jkad216-B59] Shephard HL, Parker JS, Darby P, Ainsworth CC. 2000. Sexual development and sex chromosomes in hop. New Phytol. 148:397–411. doi:10.1046/j.1469-8137.2000.00771.x. 33863027

[jkad216-B60] Sorensen A-M, Kröber S, Unte US, Huijser P, Dekker K, Saedler H. 2003. The Arabidopsis *ABORTED MICROSPORES (AMS*) gene encodes a MYC class transcription factor. Plant J. 33:413–423. doi:10.1046/j.1365-313X.2003.01644.x. 12535353

[jkad216-B61] Thomas GG, Neve RA. 1976. Studies on the effect of pollination on the yield and resin content of hops (*Humulus lupulus* L.). J Inst Brew. 82:41–45. doi:10.1002/j.2050-0416.1976.tb03720.x.

[jkad216-B62] Toth JA, Stack GM, Cala AR, Carlson CH, Wilk RL, Crawford JL, Viands DR, Philippe G, Smart CD, Rose JKC, et al 2020. Development and validation of genetic markers for sex and cannabinoid chemotype in *Cannabis sativa* L. GCB Bioenergy. 12:213–222. doi:10.1111/gcbb.12667.

[jkad216-B63] Truett GE, Heeger P, Mynatt RL, Truett AA, Walker JA, Warman ML. 2000. Preparation of PCR-quality mouse genomic DNA with hot sodium hydroxide and Tris (HotSHOT). Biotechniques. 29:52–54. doi:10.2144/00291bm09. 10907076

[jkad216-B64] Vasimuddin M, Misra S, Li H, Aluru S. 2019. Efficient architecture-aware acceleration of BWA-MEM for multicore systems. In: 2019 IEEE International Parallel and Distributed Processing Symposium (IPDPS) Rio de Janeiro, Brazil. p. 314–324. doi:10.1109/IPDPS.2019.00041.

[jkad216-B65] Verma S, Negi NP, Pareek S, Mudgal G, Kumar D. 2022. Auxin response factors in plant adaptation to drought and salinity stress. Physiol Plant. 174:e13714. doi:10.1111/ppl.13714. 35560231

[jkad216-B66] Wang J, Zhang Z. 2021. GAPIT version 3: boosting power and accuracy for genomic association and prediction. Genomics Proteomics Bioinformatics. 19:629–640. doi:10.1016/j.gpb.2021.08.005. 34492338 PMC9121400

[jkad216-B68] Xin Z, Velten JP, Oliver MJ, Burke JJ. 2003. High-throughput DNA extraction method suitable for PCR. Biotechniques. 34:820–826. doi:10.2144/03344rr04. 12703307

[jkad216-B69] Xu J, Ding Z, Vizcay-Barrena G, Shi J, Liang W, Yuan Z, Werck-Reichhart D, Schreiber L, Wilson ZA, Zhang D. 2014. *ABORTED MICROSPORES* acts as a master regulator of pollen wall formation in *Arabidopsis*. Plant Cell. 26:1544–1556. doi:10.1105/tpc.114.122986. 24781116 PMC4036570

[jkad216-B70] Yamaoka S, Nishihama R, Yoshitake Y, Ishida S, Inoue K, Saito M, Okahashi K, Bao H, Nishida H, Yamaguchi K, et al 2018. Generative cell specification requires transcription factors evolutionarily conserved in land plants. Curr Biol. 28:479–486.e5. doi:10.1016/j.cub.2017.12.053. 29395928

[jkad216-B72] Yu J, Pressoir G, Briggs WH, Vroh Bi I, Yamasaki M, Doebley JF, McMullen MD, Gaut BS, Nielsen DM, Holland JB, et al 2006. A unified mixed-model method for association mapping that accounts for multiple levels of relatedness. Nat Genet. 38:203–208. doi:10.1038/ng1702. 16380716

[jkad216-B73] Yue J, Chen Q, Wang Y, Zhang L, Ye C, Wang X, Cao S, Lin Y, Huang W, Xian H, et al 2023. Telomere-to-telomere and gap-free reference genome assembly of the kiwifruit *Actinidia chinensis*. Hortic Res. 10:uhac264. doi:10.1093/hr/uhac264. 36778189 PMC9909506

[jkad216-B76] Zhang L, Fetch T, Nirmala J, Schmierer D, Brueggeman R, Steffenson B, Kleinhofs A. 2006. *Rpr1*, a gene required for *Rpg1*-dependent resistance to stem rust in barley. Theor Appl Genet. 113:847–855. doi:10.1007/s00122-006-0342-y. 16832646

[jkad216-B74] Zhang R, Chang J, Li J, Lan G, Xuan C, Li H, Ma J, Zhang Y, Yang J, Tian S, et al 2021. Disruption of the bHLH transcription factor Abnormal Tapetum 1 causes male sterility in watermelon. Hortic Res. 8:258. doi:10.1038/s41438-021-00695-9. 34848708 PMC8632879

[jkad216-B77] Zhang S, Wu Z, Ma D, Zhai J, Han X, Jiang Z, Liu S, Xu J, Jiao P, Li Z. 2022. Chromosome-scale assemblies of the male and female *Populus euphratica* genomes reveal the molecular basis of sex determination and sexual dimorphism. Commun Biol. 5:1–16. doi:10.1038/s42003-022-04145-7. 36333427 PMC9636151

[jkad216-B75] Zhang Z, Ersoz E, Lai C-Q, Todhunter RJ, Tiwari HK, Gore MA, Bradbury PJ, Yu J, Arnett DK, Ordovas JM, et al 2010. Mixed linear model approach adapted for genome-wide association studies. Nat Genet. 42:355–360. doi:10.1038/ng.546. 20208535 PMC2931336

